# Predictors of Switching Anti-Tumor Necrosis Factor Therapy in Patients with Ankylosing Spondylitis

**DOI:** 10.1371/journal.pone.0131864

**Published:** 2015-07-15

**Authors:** Jeong-Won Lee, Ji-Hyoun Kang, Yi-Rang Yim, Ji-Eun Kim, Lihui Wen, Kyung-Eun Lee, Dong-Jin Park, Tae-Jong Kim, Yong-Wook Park, Shin-Seok Lee

**Affiliations:** Department of Rheumatology, Chonnam National University Medical School & Hospital, Gwangju, Republic of Korea; Oregon Health & Science University, UNITED STATES

## Abstract

The aim of this study was to investigate the potential predictors of switching tumor necrosis factor (TNF)-α inhibitors in Korean patients with ankylosing spondylitis (AS). The patients who had been treated with TNF-α inhibitors were divided into two groups depending on whether they had switched TNF-α inhibitors. Demographic, clinical, laboratory, and treatment data at the time of initiation of TNF-α inhibitor treatment were compared between switchers and non-switchers, and within switchers according to the reasons for switching. Of the 269 patients, 70 (23%) had switched TNF-α inhibitors once; of these, 11 switched again. The median follow-up time was 52.7 months. Three- and five-year drug survival rates were 52%/48% for infliximab, 62%/42% for etanercept, and 71%/51% for adalimumab, respectively. Switchers were more likely to be prescribed disease-modifying anti-rheumatic drugs than non-switchers. A history of joint surgery and complete ankylosis of the sacroiliac joint was more frequent in switchers. Multivariate Cox’s proportional hazard analysis showed that the use of adalimumab as the first TNF-α inhibitor was less likely to lead to switching and complete ankylosis of the sacroiliac joints was more likely to lead to switching. The principal reasons for switching were drug inefficacy and adverse events, but the differences in the clinical data of these two groups of switchers were not significant. In AS patients who are candidates for TNF-α inhibitor therapy, switching may improve the therapeutic outcome based on clinical information.

## Introduction

Ankylosing spondylitis (AS) is an inflammatory rheumatic disorder mainly affecting the axial skeleton. Inflammation of the sacroiliac joint, spine, and entheses is the main characteristic of AS and it eventually leads to ankylosis of the affected joint. Tumor necrosis factor (TNF)-α inhibitors are a major advance in the treatment of AS and their efficacy has been proven in several randomized controlled trials [1–3]. However, according to nationwide registries of the drug continuation rate in several countries, the rate of treatment failure is considerable [4–6], with drug survival in the range of 63–82%. Moreover, many national recommendations and guidelines do not address drug discontinuation or switching in AS patients initially treated with TNF-α inhibitors.

In Korea, infliximab, etanercept, and adalimumab are the commercially available TNF-α inhibitors. Etanercept for patients with AS was approved by the Korean Health Insurance Review & Assessment Service (HIRA) in May 2005, followed by infliximab in November 2006 and adalimumab in April 2007. Head-to-head trials comparing the safety and efficacy of these three TNF-α inhibitors are lacking but their efficacies are considered to be similar. Infliximab is an IgG1 chimeric monoclonal antibody with its Fab portion derived from mouse; it is administered by intravenous infusion. Both etanercept, a recombinant 75-kDa TNF receptor IgG1 fusion protein, and adalimumab, a humanized monoclonal antibody, are given by subcutaneous injection. The different molecular structures and routes of administration of these three drugs could influence both their efficacies and their association with adverse events in patients receiving them. Accordingly, patients who do not respond to a TNF-α inhibitor or suffer an adverse event during its use may benefit by switching to one of the other inhibitors.

For ethical reasons, the switching rate of TNF-α inhibitors and its effects cannot be investigated through randomized placebo-controlled studies. However, the many nationwide drug registries, such as BIOBADASER (Spanish Registry of Adverse Events of Biological Therapies in Rheumatic Diseases), BSRBR (The British Society for Rheumatology Biologics Registers), DANBIO (a nationwide registry of biological therapies in Denmark), and NOR-DMARD (Norwegian DMARD register) provide the basis for observational studies. To date, there is no well-organized registry of similar information in Asia. Therefore, in this work our primary objectives were: (1) to analyze the switching percentage, the order of switching, and the clinical characteristics of AS patients in Korea who had switched TNF-α inhibitors and 2) to identify the predictors for switching TNF-α inhibitors.

## Materials and Methods

This retrospective cohort study was conducted in a single tertiary center. Patients diagnosed with AS according to the modified New York Criteria [7] and in whom TNF-α inhibitors were first initiated between January 2002 and December 2013 were eligible. Those who received biological agents other than TNF-α inhibitors and with a follow-up of less than 3 months were excluded. The TNF-α inhibitors investigated in this study were infliximab, etanercept, and adalimumab. According to the Korean HIRA guidelines, the dose and interval of each TNF-α inhibitor are predetermined. The dose or frequency of TNF-α inhibitors was not escalated arbitrarily. Infliximab was administered as an intravenous infusion at a dose of 5 mg/kg at weeks 0, 2, and 6 and then every 6–8 weeks. Etanercept was administered as a subcutaneous injection of 25 mg twice per week or 50 mg once per week. Adalimumab was administered as a subcutaneous injection of 40 mg every other week. This study was approved by the Institutional Review Board of Chonnam National University Hospital (CNUH-2014-073), Republic of Korea. Although informed consent was waived due to retrospective study design, patient health information was de-identified prior to analysis and patient anonymity was preserved during the study period.

Patients in whom standard treatment failed and who had active disease were eligible for TNF-α inhibitor therapy, according to the Korean HIRA guideline based on ASAS consensus statement for the use of an anti-TNF agent for AS [8]. The failure of standard treatment was defined as symptom persistence despite adequate therapeutic trials of at least two nonsteroidal anti-inflammatory drugs or disease-modifying antirheumatic drugs (DMARDs) for more than 3 months and a Bath ankylosing spondylitis disease activity index (BASDAI; scale 1–10) >4 was defined as active disease. All patients underwent tuberculin skin testing and an interferon-γ-release assay to identify latent tuberculosis (TB) infection, in which case isoniazid was administered for at least 1 month before initiating TNF-α inhibitor treatment. Patients on TNF-α inhibitors were monitored using the BASDAI 3 months after the initiation of treatment and every 6 months thereafter. Response was determined using the Korean HIRA guidelines as an improvement of at least 50% or as a score of no more than 2 in BASDAI 3 months after the initiation of treatment and every 6 months thereafter. According to the HIRA guidelines, the TNF-α inhibitor must be switched if a patient does not respond to the treatment.

The term "inefficacy" defined as an improvement of less than 50% or as a score of more than 2 in BASDAI 3 months after the initiation of treatment and every 6 months thereafter. Inefficacy included a lack/loss of effect of a TNF-α inhibitor. "Switchers" denoted the group of patients who switched from the prescribed TNF-α inhibitor to one of the other two inhibitors. "Non-switchers" denoted the group of patients who either continued with the first TNF-α inhibitor or discontinued the first TNF-α inhibitor but did not receive any other biological agent.

To identify predictors for switching, patients were divided into two groups according to whether they had switched TNF-α inhibitors. The reasons for switching considered in this study were occurrence of an adverse event and inefficacy of treatment. Medical records were reviewed for information on patient age, body mass index (BMI), familial history, surgical history, marital status, education level, smoking status, and alcohol consumption at the time of initiation of TNF-α inhibitor treatment. Physical examination, laboratory findings, and imaging findings were reviewed at the time of treatment initiation and 3 months thereafter. BMI was calculated as the body weight in kilograms divided by the square of the height in meters. Peripheral arthritis was based on the clinical finding of one or more swollen and tender joints. Sacroiliitis was graded using plain radiographs according to the New York criteria [7]: grade 0: normal; grade I: some blurring of the joint margins—suspicious; grade II: minimal sclerosis with some erosion; grade III: definite sclerosis on both sides of the joint or severe erosions with widening of the joint space with or without ankylosis; grade IV: complete ankylosis. Treatment-related data were also reviewed with respect to medications and their doses. DMARDs that we investigated were the ‘conventional’ DMARDs: methotrexate, hydroxychloroquine, sulfasalazine, and leflunomide.

### Statistical analysis

Data processing and statistical analyses were performed using SPSS version 21.0 (SPSS, Chicago, IL). Continuous variables are presented as the medians and interquartile range and were compared using a Mann-Whitney U-test. Categorical variables were analyzed using a χ^2^ test or Fisher’s exact test. Univariate and multivariate Cox regression analyses with hazard ratios (HRs) were used to identify factors predicting TNF-α inhibitor switching. *P* < 0.05 was considered to indicate statistical significance.

## Results

All 269 patients with AS who participated in the study had been administered at least one TNF-α inhibitor. The median age of the patients was 40 years (range: 19–73) and 218 (81%) were males. The median duration of follow-up was 57.2 months (range: 3–143 months). Three- and five-year drug survival rates were 52%/48% for infliximab, 62%/42% for etanercept, and 71%/51% for adalimumab, respectively (S1 File).

In the case of the first TNF-α inhibitors, 70 of 269 (26%) patients were switchers and 199 of 269 (74%) patients were non-switchers. In the case of second TNF-α inhibitors, 11 of 70 (15.7%) were switchers and 59 of 70 (84.3%) were non-switchers. In the case of third TNF-α inhibitors, no patient who switched their TNF-α inhibitors has been identified to date. Among the 199 (74%) patients who did not switch first TNF-α inhibitors (non-switchers), 150 continued with the first inhibitor and 49 completely discontinued their use. Among 49 patients who discontinued TNF-α inhibitors, 34 did so due to low disease activity, 4 due to pregnancy and breast-feeding, and 2 due to economic issues. In total, nine patients discontinued their TNF-α inhibitors due to adverse events: six experienced mild injection reaction, and they maintained low disease activity even after discontinuation, two patients were discontinued due to pulmonary tuberculosis, and one patient discontinued due to congestive heart failure.

Infliximab was the first TNF-α inhibitor used in the majority of patients (n = 108, 40.1%), followed by etanercept (n = 95, 35.3%) and adalimumab (n = 66, 24.5%). Of the switchers, 38 patients (54.2%) were initially on infliximab, 26 (37.1%) were on etanercept, and 6 (8.5%) were on adalimumab. For the second TNF-α inhibitor, 13 (18.5%) patients were administered infliximab, 12 (17.1%) received etanercept, and 45 (64.2%) were treated with adalimumab (Table 1). The most common sequence of switching was infliximab to adalimumab (27 patients, 38.5%) followed by etanercept to adalimumab (18 patients, 25.7%). Only one patient (1.4%) switched from adalimumab to etanercept.

**Table 1:** Number of patients according to the course of treatment.

Course of treatment	Infliximab	Etanercept	Adalimumab	Total
1st TNF-α inhibitor	108 (40.1%)	95 (35.3%)	66 (24.5%)	269
Switching	38 (54.2%)	26 (37.1%)	6 (8.5%)	70
2nd TNF-α inhibitor	13 (18.5%)	12 (17.1%)	45 (64.2%)	70

TNF, tumor necrosis factor.

Demographic, socioeconomic, clinical, laboratory, and treatment data at the time of TNF-α inhibitor initiation were compared between switchers and non-switchers (Table 2). The first TNF-α inhibitors were used for a median of 20 months by switchers and 43 months by non-switchers. The percentage of patients who underwent joint surgery, such as synovectomy or total joint replacement, prior to the initiation of the first TNF-α inhibitor and the percentage of patients with complete ankylosis of the sacroiliac joint were higher in switchers than in non-switchers (*p* = 0.010 and *p* = 0.013). In the baseline laboratory findings, median erythrocyte sedimentation rate and C-reactive protein levels were higher in switchers than in non-switchers (*p* = 0.038 and *p* = 0.002). Methotrexate and sulfasalazine as well as DMARDs were more frequently prescribed to switchers (*p* = 0.020, *p* = 0.016 and *p* = 0.003) and the median prescribed dose of prednisolone was higher (*p* = 0.022).

**Table 2:** Baseline characteristics and laboratory findings of patients starting their first TNF-α inhibitor according to whether they subsequently switched or not.

	Non-switchers	Switchers	*p*-value
Age, years	35.0 (28.0–45.0)	36.0 (28.0–44.2)	0.998
Men	162 (81.4%)	56 (80%)	0.796
Disease duration, years	1.0 (0–5.0)	1.0 (0–6.0)	0.668
Smoking	54 (36.2%)	22 (40.7%)	0.559
Alcohol consumption	64 (42.4%)	19 (35.2%)	0.355
Family history	13 (8.3%)	4 (6.8%)	0.715
History of joint surgery	21 (12.4%)	16 (22.9%)	0.010
Marital status	50 (68.5%)	27 (64.3%)	0.644
More than 12 years of education, years	32 (47.1%)	20 (51.3%)	0.674
Duration of first TNF-α inhibitor use, months	43.0 (23.0–67.0)	20.0 (10.0–33.0)	<0.001
BMI, kg/m^2^	23.2 (21.4–25.2)	22.7 (20.3–25.3)	0.836
Uveitis	23 (12.8%)	14 (21.9%)	0.081
Peripheral arthritis	47 (23.7%)	20 (29.9%)	0.320
Complete ankylosis	52 (26.9%)	29 (43.3%)	0.013
BASDAI	6.8 (5.9–7.7)	6.9 (5.5–8.4)	0.407
WBC, 10^3^/mm^3^	7.9 (6.8–9.4)	8.1 (6.7–9.3)	0.904
Hemoglobin, g/dL	13.7 (12.2–14.7)	12.7 (11.6–14.4)	0.065
Platelets, 10^3^/mm^3^	298.0 (253.5–359.0)	313.5 (266.2–376.2)	0.172
ESR, mm/h	41.0 (21.0–76.0)	55.0 (31.0–87.5)	0.038
CRP, mg/dL	1.4 (0.6–2.8)	2.3 (1.1–5.9)	0.002
AST, U/L	20.0 (17.0–25.0)	21.0 (17.7–27.2)	0.185
ALT, U/L	16.0 (12.0–25.0)	16.0 (12.0–26.0)	0.996
Total bilirubin, mg/dL	0.55 (0.44–0.71)	0.52 (0.40–0.67)	0.317
Creatinine, mg/dL	0.70 (0.70–0.90)	0.75 (0.60–0.82)	0.929
HLA B27 positivity	172 (90.5%)	58 (89.2%)	0.807
INH prophylaxis	105 (52.8%)	29 (41.4%)	0.103
NSAIDs	105 (52.8%)	43 (61.4%)	0.210
Prednisolone	34 (17.1%)	12 (17.1%)	0.991
Methotrexate	48 (24.1%)	27 (38.6%)	0.020
Sulfasalazine	33 (16.6%)	21 (30.0%)	0.016
Any DMARDs	68 (34.3%)	38 (54.3%)	0.003
Median dose of prednisolone, mg/day	5.0 (5.0–5.0)	5.5 (5.0–16.0)	0.022
Median dose of methotrexate, mg/week	10.0 (7.5–15.0)	10.0 (7.5–15.0)	0.995
Median dose of sulfasalazine, g/day	1.0 (1.0–1.5)	1.0 (1.0–2.0)	0.976

TNF, tumor necrosis factor; BMI, body mass index; BASDAI, Bath Ankylosing Spondylitis Disease Activity Index; ESR, erythrocyte sedimentation rate; CRP, C-reactive protein; AST, aspartate aminotransferase; ALT, alanine aminotransferase; INH, isoniazid; NSAIDs, non-steroidal anti-inflammatory drugs; DMARDs, disease-modifying anti-rheumatic drugs. Continuous variables are shown as medians and interquartile range.

The principal reasons for switching were drug inefficacy (45 patients, 64.3%) and the occurrence of adverse events (18 patients, 25.7%), with infusion reaction (13 patients) being the most common among the latter. The median dosage of methotrexate at the time of TNF-α inhibitor initiation was higher in the group of patients who had switched inhibitors because of drug inefficacy than in the group reporting adverse events (7.5 mg; range 7.5–8.7 mg vs. 12.5 mg, range 7.5–15.0 mg, *p* = 0.044) (Table 3). Among the patients who had experienced an adverse event, the percentage of those with 12 or more years of education was higher than in the group with drug inefficacy (55.6% vs. 17.8%, *p* = 0.005). The same was true for patients with uveitis. C-reactive protein levels tended to be lower in the group with adverse event. We also had attempted to divide the ‘inefficacy’ group into ‘lack of efficacy’ and ‘loss of efficacy.’ Lack of efficacy was defined as initially not responding to TNF-α inhibitors within 3–4 months. Loss of efficacy was defined as an initial response to TNF-α inhibitors and a secondary loss of efficacy over 6 months. According to this definition, only 4 patients were allocated to the lack of efficacy group and 41 to the loss of efficacy group. When we analyzed the data of only the loss of efficacy group, there was no difference from the original result.

**Table 3:** Baseline characteristics of patients at the time of TNF-α inhibitor initiation according to the reason for subsequent switching.

	Adverse event (n = 18)	Inefficacy (n = 45)	*p*-value
Age, years	38.0 (30.0–46.2)	34.0 (27.5–45.5)	0.394
Males	13 (72.2%)	36 (80.0%)	0.504
Disease duration, years	1.0 (0–5.2)	1.0 (0–7.0)	0.988
Smoking	6 (33.3%)	14 (31.1%)	1.000
Alcohol consumption	8 (44.4%)	11 (24.4%)	0.138
Family history	1 (5.9%)	2 (5.4%)	0.943
History of joint surgery	3 (16.7%)	10 (22.2%)	0.741
Marital status	10 (71.4%)	15 (62.5%)	0.577
More than 12 years of education, years	10 (55.6%)	8 (17.8%)	0.005
Duration of 1st TNF α inhibitor use, months	20.5 (10.7–34.2)	21.0 (10.5–36.0)	0.605
BMI, kg/m^2^	25.1 (21.5–27.0)	22.5 (19.7–24.6)	0.066
Uveitis	6 (35.3%)	6 (14.6%)	0.085
Peripheral arthritis	6 (33.3%)	13 (30.2%)	0.812
Complete ankylosis	5 (27.8%)	21 (50%)	0.117
BASDAI	8.0 (5.4–9.0)	6.7 (5.5–7.8)	0.205
WBC, 10^3^/mm^3^	8.1 (6.5–8.6)	8.3 (7.1–9.3)	0.223
Hemoglobin, g/dL	13.0 (12.0–14.6)	12.6 (11.4–14.5)	0.429
Platelets, 10^3^/mm^3^	306.0 (251–358.5)	315.0 (269.0–391.0)	0.304
ESR, mm/h	48.5 (31.5–66.7)	52.5 (25.5–88.7)	0.926
CRP, mg/dL	1.3 (0.5–2.6)	2.8 (1.2–6.3)	0.065
AST, U/L	23.0 (16.0–34.2)	20.0 (16.5–23.5)	0.214
ALT, U/L	14.5 (11.5–36.0)	16.0 (11.5–23.5)	0.964
Total bilirubin, mg/dL	0.56 (0.43–0.70)	0.50 (0.40–0.67)	0.625
Creatinine, mg/dL	0.75 (0.67–0.82)	0.70 (0.60–0.85)	0.681
HLA B27 positivity	15 (83.3%)	37 (92.5%)	0.300
INH prophylaxis	10 (55.6%)	18 (40%)	0.265
NSAIDs	8 (44.4%)	29 (64.4%)	0.149
Prednisolone	1 (5.6%)	10 (22.2%)	0.147
Methotrexate	5 (27.8%)	19 (42.2%)	0.290
Sulfasalazine	6 (33.3%)	12 (26.7%)	0.597
Any DMARDs	7 (38.9%)	27 (60%)	0.133
Median dose of methotrexate, mg/week	7.5 (7.5–8.7)	12.5 (7.5–15.0)	0.044
Median dose of sulfasalazine, g/day	1.0 (1.0–2.0)	1.0 (1.0–2.0)	0.898

TNF, tumor necrosis factor; BMI, body mass index; BASDAI, Bath Ankylosing Spondylitis Disease Activity Index; ESR, erythrocyte sedimentation rate; CRP, C-reactive protein; AST, aspartate aminotransferase; ALT, alanine aminotransferase; INH, isoniazid; NSAIDs, non-steroidal anti-inflammatory drugs; DMARDs, disease-modifying anti-rheumatic drugs.

Continuous variables are shown as medians and interquartile range.

Multivariate Cox’s proportional hazard models were used to assess the reliability of the switching predictors identified (Table 4). The use of adalimumab as the first TNF-α inhibitor was less likely to lead to switching (HR 0.323, 95% Cl 0.134–0.779), whereas complete ankylosis evident on radiographs of the sacroiliac joint was more likely to lead to switching (HR 1.868, 95% Cl 1.128–3.095). History of joint surgery, hemoglobin level, C-reactive protein level, and concomitant DMARD therapy were not statistically significant predictors of switching.

**Table 4:** Predictors of TNF-α inhibitor switching.

Variable	Univariate analysis		Multivariate analysis	
	HR (95% CI)	*p v*alue	HR (95% CI)	*p* value
First TNF-α inhibitor				
Inflixmab				
Etanercept	0.693 (0.419–1.146)	0.153	0.626 (0.361–1.054)	0.077
Adalimumab	0.257 (0.109–0.610)	0.002	0.323 (0.134–0.779)	0.012
History of joint surgery	2.070 (1.182–3.625)	0.011	1.682 (0.934–3.031)	0.083
Complete ankylosis	1.857 (1.142–3.020)	0.013	1.868 (1.128–3.095)	0.015
Hemoglobin, g/dL	0.884 (0.778–1.005)	0.060	0.925 (0.779–1.099)	0.374
Platelets, 10^3^/mm^3^	1.001 (0.999–1.004)	0.329		
ESR, mg/dL	1.001 (0.998–1.003)	0.564		
CRP, mg/dL	1.081 (1.013–1.153)	0.018	1.019 (0.930–1.116)	0.693
Methotrexate	1.475 (0.907–2.400)	0.117		
Sulfasalazine	1.581 (0.939–2.660)	0.085		
Any DMARDs	1.698 (1.057–2.729)	0.029	1.281 (0.765–2.146)	0.346
Median dose of prednisolone, mg/day	1.024 (0.950–1.104)	0.528		

ESR, erythrocyte sedimentation rate; CRP, C-reactive protein; DMARDs, disease-modifying anti-rheumatic drugs.

The group of switchers due to drug inefficacy was compared with the group of non-switchers. The percentages of patients who underwent joint surgery before starting their first TNF-α inhibitor and of patients with complete ankylosis of the sacroiliac joint were higher in the inefficacy group than in the non-switchers group (12.4% vs. 26.3%, *p* = 0.030 and 26.9% vs. 50.0%, *p* = 0.003). Median C-reactive protein levels were also higher in the inefficacy group (*p* = 0.001). Methotrexate and any DMARD were more frequently prescribed to patients in the inefficacy group than to those in the non-switchers group (*p* = 0.014 and *p* = 0.001). Complete ankylosis of the sacroiliac joint was more likely to lead to switching in the inefficacy group, based on an analysis of the data by multivariate Cox’s proportional hazard analysis (HR 2.087, *p* = 0.001). These results are similar to those between switchers and non-switchers.

## Discussion

In this study, 23% of AS patients treated with one of the three most commonly used TNF-α inhibitors had switched to another. The principle reason for switching was inefficacy. The use of adalimumab as the first TNF-α inhibitor was less likely to lead to switching and complete ankylosis in the sacroiliac joint was more likely to lead to switching.

Our results are in line with those of nationwide registries in many other countries, in which 15–30% of AS patients had switched TNF-α inhibitors. In the Norwegian NOR-DMARD registry, the switching rate of AS patients within a 9-year period was 14.9% (77/514) [9]. In the Danish DANBIO registry, among AS patients treated with TNF-α inhibitors and followed for 10 years 30% had switched once and 10% had switched twice [10]. French registries report switching rates of 26% (99/377) and 32% (72/222) in patients with spondyloarthropathy [11, 12]. In the Spanish BIOBADASER registry, among 4,706 patients with chronic arthritis, including rheumatoid arthritis (RA), psoriatic arthritis (PsA), and AS, 488 had been treated with more than one TNF-α inhibitor over a 4-year period [13]. Similarly, an observational study conducted in Austria investigated TNF-α inhibitor switching in patients with chronic arthritis, including RA, PsA, and AS; 38% of patients with chronic arthritis had received more than one TNF-α inhibitor [14]. Thus, the switching rate of European and Asian patients seems to be similar.

Infliximab was the most frequently used agent among the three TNF-α inhibitors considered in this study, followed by adalimumab and etanercept. The most common sequence of switching was infliximab to adalimumab. Infliximab was the first TNF-α inhibitor introduced for the treatment of RA in Korea. However, it had been used for the treatment of AS even before receiving the approval of the Korean HIRA for this purpose. The early off-label use of infliximab probably explains why patients with more severe AS were initially treated with infliximab than with the other two TNF-α inhibitors. Accordingly, preference and accessibility may influence the decision to switch from infliximab to another agent. We analyzed baseline characteristics according to TNF-α inhibitor, additionally. Age, ESR, and CRP, at the time of initiation of TNF-α inhibitors, were highest in the infliximab group. Disease duration was longest in infliximab group but there was no statistically significant difference among the three agents. Concomitant uses of DMARDs and MTX were lowest in the adalimumab group.

In several reports, TNF-α inhibitors were discontinued because of the development of anti-drug antibodies, which occurs more often with infliximab than with adalimumab. This difference can be explained by the structures of these inhibitors. Infliximab, a chimeric monoclonal antibody, has a murine component and therefore a greater likelihood of inducing anti-drug antibodies than adalimumab, a humanized monoclonal antibody [15]. Because anti-drug antibodies neutralize TNF-α inhibitors, their presence is associated with lower trough concentrations of these drugs [16] and therefore, eventually, with a diminished clinical response [17]. This would account for the more frequent switching from infliximab to one of the other two TNF-α inhibitors, as determined in this study. According to one report, 72.7% of patients who experienced an infusion reaction to TNF-α inhibitor showed high anti-drug antibody titers [18]. Among the switchers in our study, 13 patients switched because of an infusion reaction, 10 of whom had been administered infliximab as the first TNF-α inhibitor. Thus, it is likely that the high frequency of infusion reactions in patients with infliximab reflects the development of anti-drug antibodies, although neither anti-drug antibody titers nor drug concentrations were measured in this study.

Patients treated with adalimumab as the first TNF-α inhibitor were less likely to switch to another inhibitor. In the BIOBADASER registry, the use of adalimumab was a protective factor for drug discontinuation related to an adverse event in patients with RA and spondyloarthropathy [19]. Although that registry has not been used in a study to identify predictors for switching TNF-α inhibitors, the result support our findings. As selection bias exists in our retrospective study, we performed Cox’s proportional regression analysis using propensity score matching after adjusting for age, gender, disease duration, and BASDAI (Table E in S1 File). We found that adalimumab was still less likely to lead to switching even after propensity score matching.

In the current study, patients who had complete ankylosis of the sacroiliac joint when TNF-α inhibitor treatment was initiated were likely to switch to another inhibitor. This association between grade of sacroiliitis and likelihood of switching is a novel finding of our study. The radiologic response of patients with AS to TNF-α inhibitors can be measured using the Ankylosing Spondylitis spine Magnetic Resonance Imaging-activity (ASspiMRI-a) system, the Berlin modification of ASspiMRI-a and of the Spondyloarthritis Research Consortium of Canada (SPARCC) scoring system. These scoring systems use magnetic resonance imaging to measure vertebral inflammation [20]. In our study, sacroiliitis was assessed on plain radiography, which, similar to computed tomography, detects structural changes in the sacroiliac joint, such as sclerosis, erosions, and ankylosis. Because severe structural changes are indicative of highly advanced disease, our results suggest that advanced patients do not respond well to TNF-α inhibitors. When the analysis was limited to switchers due to inefficacy to TNF-α inhibitors, the results were similar to those of the total group of switchers. In the inefficacy group, complete ankylosis of the sacroiliac joint was more likely to lead to switching.

There are several potential limitations to the interpretation of our data. The first is the study design. As ours was an observational study, the choice of TNF-α inhibitors was not randomized but was instead affected by factors such as physician preference, concerns associated with the introduction of a novel agent, and the route of drug administration. The second limitation is the nature of our patient series. Patients were selected from a single tertiary center located within the local community. Thus, the patients’ living standards and their ability to access the center may have biased enrollment to favor patients with more severe disease. Third, inefficacy could not be further distinguished as lack of effect vs. loss of effect. Fourth, we did not measure anti-drug antibody levels to predict therapeutic response in these patients. Finally, we did not measure functional status of patient such as Bath Ankylosing Spondylitis Functional Index.

In conclusion, 70 (26%) of the 269 AS patients enrolled in our study had switched TNF-α inhibitors during the 12 years of follow-up. The use of adalimumab as the first TNF-α inhibitor was less likely to lead to switching and complete ankylosis of the sacroiliac joint at the time of TNF-α inhibitor initiation was more likely to lead to switching. In AS patients who are candidates for TNF-α inhibitor therapy, switching is considered to be an important treatment option, which highlights the different molecular structures and routes of administration of TNF-α inhibitors.

## Supporting Information

S1 File Supporting information Figure and Tables. Survival curves of the three TNF-α inhibitors (Fig. A). Survival rates of the three TNF-α inhibitors (Table A). Predictors of TNF-α inhibitor switching excluding the 9 patients who discontinued TNF-α inhibitors due to adverse events (Table B). Univariate logistic analysis used to evaluate the predictors of complete discontinuation of TNF-α inhibitors (Table C). Comorbid conditions of patients according to whether they subsequently switched or not (Table D). Cox proportional regression analysis used to evaluate predictors of TNF-α inhibitor switching after adjusted for age, gender, disease duration, and BASDAI using propensity score matching (Table E). Baseline characteristics and laboratory findings of patients starting their first TNF-α inhibitor according to TNF-α inhibitor using ANOVA test (Table F). Baseline characteristics and laboratory findings of patients starting their first TNF-α inhibitor according to TNF-α inhibitor using χ^2^ test (Table G).
(PDF)
