# Supplementary material for: Predictors of Switching Anti-Tumor Necrosis Factor Therapy in Patients with Ankylosing Spondylitis
Source: PLoS One. 2015 Jul 15;10(7):e0131864. doi: 10.1371/journal.pone.0131864 (PMC4503780; doi:10.1371/journal.pone.0131864)
Supplement: S1 File — Survival curves of the three TNF-α inhibitors (Fig. A). Survival rates of the three TNF-α inhibitors (Table A). Predictors of TNF-α inhibitor switching excluding the 9 patients who discontinued TNF-α inhibitors due to adverse events (Table B). Univariate logistic analysis used to evaluate the predictors of complete discontinuation of TNF-α inhibitors (Table C). Comorbid conditions of patients according to whether they subsequently switched or not (Table D). Cox proportional regression analysis used to evaluate predictors of TNF-α inhibitor switching after adjusted for age, gender, disease duration, and BASDAI using propensity score matching (Table E). Baseline characteristics and laboratory findings of patients starting their first TNF-α inhibitor according to TNF-α inhibitor using ANOVA test (Table F). Baseline characteristics and laboratory findings of patients starting their first TNF-α inhibitor according to TNF-α inhibitor using χ2 test (Table G). (PDF) [file pone.0131864.s001.pdf]

Figure A. Survival curves of the three TNF-α inhibitors.

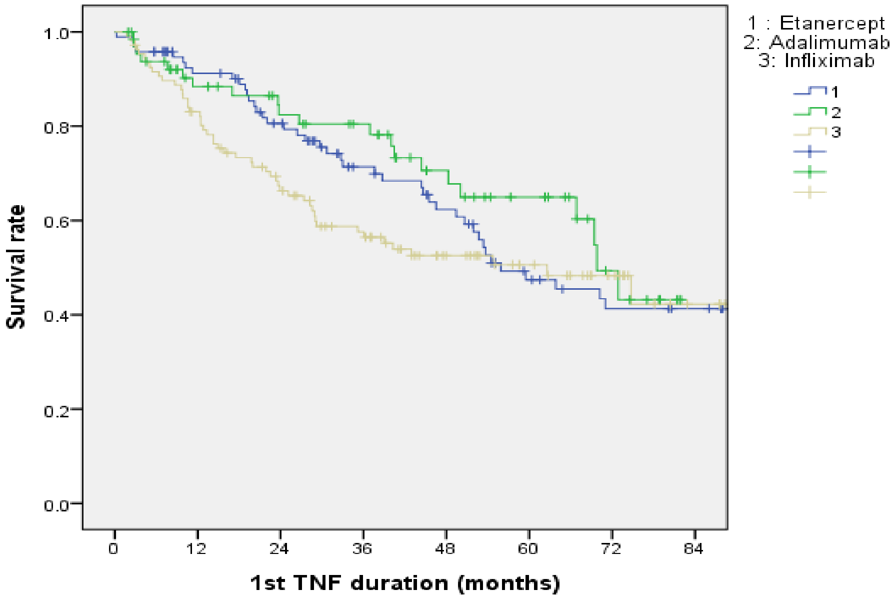

Table A. Survival rates of the three TNF-α inhibitors.

	Infliximab (%)	Etanercept(%)	Adalimumab(%)
1 year	66	81	83
2 year	58	71	81
3 year	52	62	71
4 year	50	47	64
5 year	48	42	51
6 year	42	42	40
7 year	42	42	

Table B. Predictors of TNF-α inhibitor switching excluding the 9 patients who discontinued TNF-α inhibitors due to adverse events.

Variable	Multivariate analysis	
	HR (95% CI)	*p value*
First TNF-α inhibitor		
Inflixmab		
Etanercept	0.729 (0.419-1.269)	0.263
Adalimumab	0.410 (0.169-0.996)	0.049
History of joint surgery	1.775 (0.977-3.225)	0.060
Complete ankylosis	1.835 (1.080-3.118)	0.025

Table C. Univariate logistic analysis used to evaluate the predictors of complete discontinuation of TNF-α inhibitors.

Variable	Univariate analysis	
	HR (95% CI)	*p* value
Age, years	0.981 (0.957-1.006)	0.142
Males	1.740 (0.921-3.287)	0.088
Disease duration, years	0.942 (0.863-1.029)	0.188
Smoking	1.593 (0.834-3.043)	0.158
Alcohol drinking	0.824 (0.432-1.571)	0.557
Family history	0.989 (0.304-3.217)	0.985
History of joint surgery	1.005 (0.398-2.539)	0.991
Marital status	1.747 (0.575-5.312)	0.325
More than 12 years of education, years	2.170 (0.770-6.112)	0.143
BMI, kg/m^2	0.929 (0.797-1.083)	0.345
Uveitis	1.838 (0.769-4.392)	0.171
Peripheral arthritis	0.816 (0.407-1.634)	0.565
Sacroiliitis Gr IV	0.912 (0.464-1.793)	0.789
BASDAI	0.923 (0.745-1.144)	0.464
WBC, $10^3/mm^3$	1.000 (1.000-1.000)	0.240
Hemoglobin, g/dL	1.011 (0.859-1.190)	0.895
Platelets, $10^3/mm^3$	0.998 (0.995-1.002)	0.359
ESR, mg/dL	1.002 (1.000-1.004)	0.117
CRP, mg/dL	0.993 (0.897-1.100)	0.898
AST, U/L	0.966 (0.924-1.010)	0.127

ALT, U/L	0.981 (0.957-1.006)	0.131
Total bilirubin, mg/dL	1.405 (0.391-5.041)	0.602
Creatinine, mg/dL	0.650 (0.115-3.679)	0.627
HLA B27 positivity	0.584 (0.247-1.380)	0.221
INH prophylaxis	0.958 (0.546-1.681)	0.882
NSAIDs	0.760 (0.434-1.332)	0.339
Prednisolone	0.569 (0.254-1.273)	0.170
Methotrexate	0.887 (0.474-1.659)	0.707
Sulfasalazine	0.506 (0.200-1.279)	0.150
Any DMARDs	0.837 (0.463-1.512)	0.555
Mean dose of prednisolone, mg/day	0.947 (0.817-1.097)	0.467
Mean dose of MTX, mg/week	0.953 (0.805-1.128)	0.575
Mean dose of SSZ, g/day	1.001 (0.999-1.002)	0.489
First TNF-α inhibitor		
Inflixmab		
Etanercept	0.879 (0.443-1.745)	0.713
Adalimumab	1.195 (0.604-2.366)	0.609

Table D. Comorbid conditions of patients according to whether they subsequently switched or not.

	Non-switchers	Switchers	χ^2
Comorbidity	22 (11.1%)	14 (20.0%)	0.059
Hypertension	11 (5.5%)	7 (10.0%)	0.198
Diabetes mellitus	11 (5.5%)	4 (5.7%)	1.000
Ischemic heart disease	2 (1.0%)	0	1.000
Chronic liver disease	2 (1.0%)	2 (2.9%)	0.278

Table E. Cox proportional regression analysis used to evaluate predictors of TNF-α inhibitor switching after adjusted for age, gender, disease duration, and BASDAI using propensity score matching.

Variable	Multivariate analysis	
	HR (95% CI)	*p* value
First TNF-α inhibitor		
Inflixmab		
Etanercept	0.671 (0.346-1.303)	0.239
Adalimumab	0.258 (0.104-0.639)	0.003

Table F. Baseline characteristics and laboratory findings of patients starting their first TNF-α inhibitor according to TNF-α inhibitor using ANOVA test.

Variables	Infliximab (*n* = 108)	Etanercept (*n* = 95)	Adalimumab (*n* = 66)	F	p-value
Age, years	38.6±12.3*	34.5±11.5*	37.2±11.7	3.049	0.049
Disease duration, years	3.5±4.5	3.0±3.9	3.4±4.2	0.482	0.618
Duration of first TNF-α inhibitor use, months	36.3±26.0	44.8±30.3	38.0±26.1	2.603	0.076
BMI, kg/m^2	24.0±3.6	23.2±3.8	21.9±1.9	2.394	0.096
BASDAI	6.8±1.4	7.0±1.3	6.8±1.3	0.516	0.597
WBC, $10^3/mm^3$	8.2±2.1	8.3±2.6	8.2±1.7	0.032	0.968
Hemoglobin, g/dL	12.9±1.9*	13.6±1.7*	13.4±1.8	3.783	0.024
Platelets, $10^3/mm^3$	331.0±114.1	310.1±78.4	302.6±80.0	2.076	0.127
ESR, mm/h	70.4±108.3*	44.3±31.4*	50.9±35.2	3.378	0.036
CRP, mg/dL	3.3±3.5*	2.5±2.7	2.0±2.2*	4.097	0.018
AST, U/L	24.4±23.7	23.3±13.8	23.3±11.6	0.117	0.890
ALT, U/L	21.0±15.9	22.3±23.0	23.6±20.4	0.372	0.690
Total bilirubin, mg/dL	0.57±0.21	0.58±0.24	0.58±0.20	0.052	0.950
Creatinine, mg/dL	0.75±0.30	0.79±0.16	0.75±0.21	0.855	0.426
Mean dose of prednisolone,	1.0±3.5	1.2±2.6	0.8±1.7	0.500	0.607

mg/day					
Mean dose of methotrexate, mg/week	11.9±3.5^{+}	8.9±2.4*$^{+}$	12.1±4.0*	8.802	<0.001
Mean dose of sulfasalazine, g/day	1.4±0.5*	1.1±0.5*	1.3±0.5	3.497	0.038

*, + $p < 0.05$ by Tukey and Tamhane tests, respectively.

Table G. Baseline characteristics and laboratory findings of patients starting their first TNF-α inhibitor according to TNF-α inhibitor using χ^2 test.

Variable	Infliximab (n=108)	Etanercept (n=95)	Adalimumab (n=66)	χ^2
Male	84 (77.8%)	85 (89.5%)	49 (74.2%)	0.028
Smoking	25 (30%)	35 (44.3%)	16 (37.2%)	0.214
Alcohol	33 (39.8%)	30 (38.0%)	20 (46.5%)	0.646
Family history	9 (10.1%)	5 (6.3%)	3 (6.3%)	0.592
History of joint surgery	11 (10.2%)	22 (23.2%)	4 (6.1%)	0.003
Marital status	38 (71.7%)	30 (63.8%)	9 (60.0%	0.584
More than 12 years of education	24 (50.0%)	23 (51.1%)	5 (35.7%)	0.582
Uveitis	11 (10.9%)	14 (16.7%)	12 (20.3%)	0.245
Peripheral arthritis	26 (24.5%)	24 (25.8%)	17 (25.8%)	0.974
Complete ankylosis	30 (29.1%)	31 (33.7%)	20 (30.8%)	0.787
HLA B27 positivity	87 (87%)	84 (91.3%)	59 (93.7%)	0.275
INH prophylaxis	50 (46.3%)	51 (53.7%)	33 (50.0%)	0.773
NSAIDs	52 (48.1%)	61 (64.2%)	35 (53.0%)	0.067
Prednisolone	13 (12.0%)	20 (21.1%)	13 (19.7%)	0.191
Methotrexate	31 (28.7%)	35 (36.8%)	9 (13.6%)	0.005
Sulfasalazine	18 (16.7%)	26 (27.4%)	10 (15.2%)	0.085
Any DMARDs	40 (37.0%)	48 (51.1%)	18 (27.3%)	0.008

DMARDs, disease-modifying anti-rheumatic drugs.
